# Comparison of ridge mapping using bone caliper, stone cast, CBCT and direct measurement in treatment planning of dental implants

**DOI:** 10.6026/973206300221602

**Published:** 2026-03-31

**Authors:** Apurv Ghogrey, Sanjeev Singh, Saumya Sharma, Priyabrata Jena, Arpana Verma, Medhavi Sahu

**Affiliations:** 1 Department of Prosthodontics and Crown and Bridge, Maitri College of Dentistry and Research Centre, Anjora, Durg, Chhattisgarh, India

**Keywords:** Alveolar ridge, CBCT, direct measurements, split cast, bone caliper

## Abstract

Adequate bone volume ensures implant stability so this study was done to evaluate the accuracy of ridge mapping using a bone caliper, stone cast, CBCT and direct measurement for alveolar ridge width determination in dental implant treatment planning. The study included 25 edentulous patients (aged 30-55, both genders). Buccolingual ridge width was assessed by Ridge Mapping, Split Cast and CBCT. Each technique was compared with direct measurement via surgical flap exposure (Gold Standard). Four sets of readings were recorded. Statistical analysis included mean, standard deviation and two-way ANOVA test. No significant differences were found among the three methods. Ridge mapping by bone caliper was most reliable, followed by CBCT and split cast technique. All three methods are equivalent to the surgical gold standard and can be used interchangeably.

## Background:

Evaluating alveolar ridge dimensions is crucial for successful implant therapy, as adequate bone volume ensures implant stability and function [1]. Pre-surgical assessment of the alveolar bone is essential for accurate diagnosis and treatment planning. Visual inspection and conventional 2D radiographs are insufficient for determining buccolingual ridge width, as they do not reveal ridge thickness [2]. Advanced imaging techniques like CBCT offer detailed, three- dimensional visualization of bone morphology and are particularly useful in areas with minimal resorption or near vital structures like the maxillary sinus or inferior alveolar nerve [3]. However, due to higher cost and radiation exposure, their use should be carefully justified [2]. Ridge mapping is a simple, radiation-free, chair-side technique that provides immediate information about ridge width. If proven accurate, it could reduce or replace the need for CBCT in routine cases [4]. Direct measurement via surgical exposure remains the gold standard for accuracy and is also performed chair-side without radiation [5]. Though it involves flap elevation, it offers reliable, immediate results. Overall, both ridge mapping and direct measurement are practical alternatives for evaluating ridge width in implant planning [6]. Therefore, it is of interest to compare of ridge mapping using bone caliper, stone cast, CBCT and direct measurement in treatment planning of dental implants.

## Materials and Methodology:

The study, conducted at Department of Prosthodontics and Crown and Bridge, MCDRC, Durg, involved 25 edentulous sites patients requiring dental implants. Alveolar ridge width was measured at 4mm, 7mm and 10mm from the ridge crest using Ridge Mapping, Split Cast technique and CBCT, guided by a stent. Surgical exposure served as the gold standard. Measurements were analyzed using descriptive statistics, two-way ANOVA and Tukey's post-hoc test to assess accuracy against the gold standard.

## Ridge mapping using bone caliper:

A clear acrylic stent was fabricated using self-cure denture base resin to cover the edentulous ridge. Guide holes (1 mm diameter) were drilled at 4 mm, 7 mm and 10 mm from the crest. After local anesthesia, the stent was placed intraorally and caliper tips were inserted through the guide holes to contact the bone. This method is minimally invasive and radiation-free.

## Split cast technique:

Mandibular impressions were made with irreversible hydrocolloid and poured in Type III gypsum. An acrylic stent with guide holes marked at 4, 7 and 10 mm was used. After anesthesia, mucosal thickness was measured with a periodontal probe through the stent. The cast was sectioned and ridge width was calculated using soft tissue measurements and cast markings.

## CBCT assessment:

CBCT scans were performed in the Oral Medicine and Radiology Department using the Genoray-Papaya 3D Plus system with patients wearing a stent containing Gutta-percha markers at 4, 7 and 10 mm from the ridge crest. Scans were done with the Frankfurt plane parallel to the floor and ridge dimensions were assessed using Theia software.

## Direct surgical measurement:

Under local anesthesia, a mucoperiosteal flap was raised and ridge width was measured at 4 mm, 7 mm and 10 mm on exposed bone using a caliper through surgical stent guide holes. Sutures were placed post-measurement. This method is the clinical gold standard for bone width assessment.

## Results:

A total of 25 edentulous mandibular sites with adequate ridge dimensions were analyzed. Alveolar ridge width was quantitatively assessed using Two-Way ANOVA. Tables 1 and 2 (see PDF) present the comparative mean ridge width evaluation. Two-Way ANOVA: According to the results obtained from the study Mean and standard deviation of alveolar ridge dimensions obtained from all the four techniques at all points is shown in Table 1 (see PDF). Table 2 (see PDF) presents Tukey's post hoc test results for multiple comparisons of alveolar ridge dimensions at 4, 7 and 10 mm. The analysis revealed no statistically significant differences among Groups I, II, III and IV.

## CBCT versus surgical exposure:

Mean differences - 0.701 mm (4 mm), 0.584 mm (7 mm), 0.768 mm (10 mm).

## Ridge mapping versus surgical exposure:

0.08 mm (4 mm), 0.12 mm (7mm), 0.24 mm (10 mm)

## Split cast versus surgical exposure:

0.84 mm (4 mm), 0.68 mm (7 mm), 0.76 mm (10 mm)

Graph 2 shows mean and standard deviation of alveolar ridge width dimensions for all four techniques.

## Two-way ANOVA:

The inference of the present study indicates that ridge mapping, CBCT measurements and split cast technique, when compared with the gold standard surgical open method, showed ridge mapping to be closest for detecting residual alveolar ridge width in implant treatment planning.

## Discussion:

Accurate diagnosis and thorough treatment planning are essential in implant dentistry, with success depending on alveolar ridge morphology and proper implant positioning [7]. Assessing ridge width is crucial, as bone quantity, quality and visualization of anatomical structures determine implant success. The conventional ridge mapping techniques have been replaced by more advanced and latest bone imaging techniques [8]. Cone Beam Computed Tomography (CBCT) has emerged as a preferred imaging technique, offering detailed three-dimensional visualization at relatively low radiation doses, crucial for implant placement in complex areas [9]. The choice of implants will depend on the quantity and quality of bone in terms of their number, diameter, length and kind [10]. Various methods have been used to assess ridge width, including ridge mapping, CT, CBCT, split cast and direct caliper measurement. Wilson [11] and Traxler *et al.* [12] have advocated for this strategy, claiming that it is a reliable and appropriate way to assess the accuracy of potential implant sites. The lack of radiation exposure to the patient and the simplicity of the ridge mapping approach are its benefits [12]. Conventional panoramic and periapical radiographs lack cross-sectional detail and are inadequate for implant site assessment [13, 14]. Ridge mapping with a bone caliper is effective for linear ridge measurements, [15] and our findings showed no significant difference between ridge mapping, CBCT and Split Cast measurements compared with direct surgical measurements, the gold standard [16].

## Conclusion:

The width of the ridge evaluated by ridge mapping with a bone caliper was closest to the gold standard of surgical flap exposure, followed by CBCT ridge mapping and cast ridge mapping. This study concluded that all three techniques are equivalent to surgical flap exposure and can be used interchangeably.
